# The GIN-McMaster guideline tool extension for the integration of quality improvement and quality assurance in guidelines: a description of the methods for its development

**DOI:** 10.1016/j.jclinepi.2022.04.002

**Published:** 2023-02

**Authors:** Thomas Piggott, Miranda W. Langendam, Elena Parmelli, Jan Adolfsson, Elie A. Akl, David Armstrong, Jeffrey Braithwaite, Romina Brignardello-Petersen, Jan Brozek, Markus Follmann, Ina Kopp, Joerg J. Meerpohl, Luciana Neamtiu, Monika Nothacker, Amir Qaseem, Paolo Giorgi Rossi, Zuleika Saz-Parkinson, Philip J. van der Wees, Holger J. Schünemann

**Affiliations:** a Department of Health Research Methods, Evidence, and Impact, McMaster University, Hamilton, Ontario, Canada; b Department of Epidemiology, and Data Science, Amsterdam UMC, University of Amsterdam, Amsterdam of Public Health Research Instute, Amsterdam, The Netherlands; c European Commission, Joint Research Centre (JRC), Ispra, Italy; d Swedish Agency for Health Technology Assessment and Assessment of Social Services, Sweden & The Department of Clinical Science, Intervention and Technology, Karolinska Institutet, Stockholm, Sweden; e American University of Beirut, Beirut, Lebanon; f Farncombe Family Digestive Health Research Institute, McMaster University, Hamilton, Ontario, Canada; g Department of Medicine, Hamilton, McMaster University, Hamilton, Ontario, Canada; h Australian Institute of Health Innovation, Level 6, 75 Talavera Rd, Macquarie University, Sydney, Australia 2109; i German Cancer Society, Berlin, Germany; j Institute for Medical Knowledge Management, Association of the Scientific Medical Societies, (AWMF-IMWi), c/o Philipps-University, Marburg, Germany; k Institute for Evidence in Medicine, Medical Center and Faculty of Medicine, University of Freiburg, Freiburg, Germany; l Cochrane Germany, Cochrane Germany Foundation, Freiburg, Germany; m American College of Physicians, Philadelphia, PA, USA; n Epidemiology Unit, Azienda Unità Sanitaria Locale - IRCCS di Reggio Emilia, Reggio Emilia, Italy; o Radboud University Medical Center, Radboud Institute for Health Sciences, IQ Healthcare and Department of Rehabilitation, The Netherlands; p Department of Biomedical Sciences Humanitas University Via Rita Levi Montalcini 4, 20090 Pieve Emanuele, Milano, Italy

**Keywords:** Guidelines, Health decision-making, Quality improvement, Quality assurance, Performance monitoring, Checklist, GRADE, GIN-McMaster Checklist

## Abstract

**Background and Objectives:**

Our objective was to develop an extension of the widely used GIN-McMaster Guideline Development Checklist and Tool for the integration of quality assurance and improvement (QAI) schemes with guideline development.

**Methods:**

We used a mixed-methods approach incorporating evidence from a systematic review, an expert workshop and a survey of experts to iteratively create an extension of the checklist for QAI through three rounds of feedback. As a part of this process, we also refined criteria of a good guideline-based quality indicator.

**Results:**

We developed a 40-item checklist extension addressing steps for the integration of QAI into guideline development across the existing 18 topics and created one new topic specific to QAI. The steps span from ‘organization, budget, planning and training’, to updating of QAI and guideline implementation.

**Conclusion:**

The tool supports integration of QAI schemes with guideline development initiatives and it will be used in the forthcoming integrated European Commission Initiative on Colorectal Cancer. Future work should evaluate this extension and QAI items requiring additional support for guideline developers and links to QAI schemes.

**What is new?**

**Key findings**
• Quality Assurance and Improvement (QAI), in particular quality indicators, are usually based on guideline recommendations. Ideally, QAI would be systematically integrated into guideline development initiatives to improve their coordination.

**What this adds to what is known?**
• The GIN-McMaster Guideline Development Checklist has been utilized widely, but until recently did not incorporate considerations for QAI.
• Integrated guidelines and QAI should begin with considerations at the initial planning stage and be incorporated throughout the process.

**What is the implication, what should change now?**
• This checklist extension is now ready to help support integrated guideline and QAI efforts and ensure QAI schemes are based on the evidence that underpin guideline recommendations.

## 1 Background

Guidelines are used by organizations worldwide to provide recommendations to health care providers, patients and the public and other decision-makers. Guidelines and quality indicators can be complementary, guidelines need quality indicators for monitoring their implementation, and quality assurance (QA) schemes need to be based on evidence from guideline recommendations. Reporting or evaluation standards have been developed for guideline-based performance measures [[1], [2], [3]]. These nine proposed standards for guidelines addressing performance measures include the selection of guidelines and recommendations, practice testing of performance measures, and review and evaluation. However, in practice there rarely is a collaboration between developers of guidelines and quality indicators. This lack of collaboration, at least in part, may result from the lack of processes involved in guideline-based quality indicator or quality assurance scheme development [4,5]. In addition, methods or frameworks outlining the steps required for closer integration of guidelines and quality assurance schemes are not available [4].

While significant research attention has emerged since the Donabedian model on quality originated in 1966, there was striking inconsistency in the definition of a quality indicator [6,7]. To overcome this limitation, we have provided an overarching definition of a quality indicator as a construct used “guide to monitor, evaluate, and improve the quality of care” [8]. We have also presented linked definitions of performance measure (“tools that quantify or describe measurable elements of practice performance”) and performance indicator (“quantifiable and measurable units or scores of practice”) to support clarity for this field of science [8].

Quality improvement is defined by Batalden and Davidoff as “the combined and unceasing efforts of everyone—healthcare professionals, patients and their families, researchers, payers, planners, and educators—to make changes that will lead to better patient outcomes (health), better system performance (care), and better professional development” [9]. ISO 9000:2015 defines QA as quality management that is directed at creation of trust that requirements around quality are satisfied [10]. quality improvment may refer to the process of improving quality, while QA often refers to an accreditation scheme or other system. In this paper, we refer to both quality improvement and QA, which, albeit differently defined above, are used often interchangeably with converging aims relating to guideline development.

The Guidelines International Network (GIN)-McMaster guideline development checklist (GDC) for guideline developers has become the standard for considering all aspects in guideline development and provided the model for many guidelines or guideline manuals [[11], [12], [13], [14], [15], [16]]. The checklist has been complemented by an extension for rapid guidelines [17] and is currently expanded for optimal stakeholder involvement in guidelines [18]. The GDC also forms the backbone to the new INGUIDE guideline development certification and credentialing program (inguide.org) and is integrated in GRADE's official app GRADEpro (www.gradepro.org).

In this article, we describe the methods for the development of the GIN-McMaster Guideline Development Checklist extension for quality assurance and improvement (QAI) that was recently published and is available online at: https://heigrade.mcmaster.ca/guideline-development/checklist-extension-for-QAI [19]. We conducted this work to create an approach for the forthcoming coordinated guideline and QAI development effort of the European Commission Initiative on Colorectal Cancer (ECICC) and updates of its initiative on breast cancer [20,21]. It can serve as model for other guidelines.

## 2 Methods

### 2.1 Overview

**Fig. 1:**
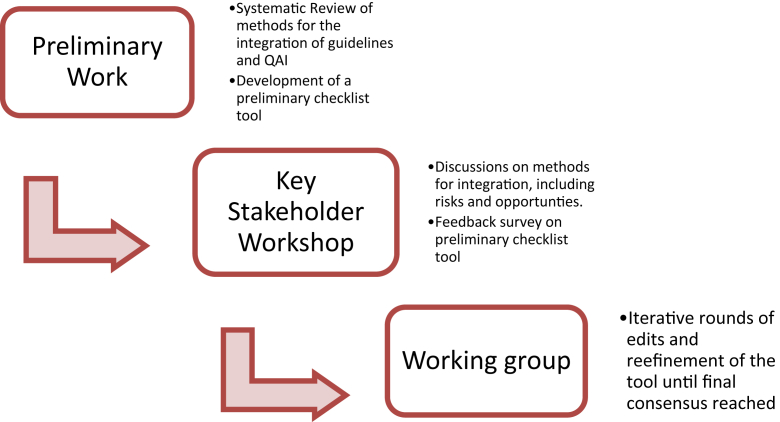
Overview of methods for the development of the GIN-McMaster Guideline Tool Extension for the Integration of Quality Improvement and Quality Assurance in Guidelines.

The integrated methodological framework for the forthcoming ECICC was the impetus for this research [4,5,8]. The approach to developing this extension is similar to that used for the GDC extension for rapid guidelines, including a systematic review of integrated guideline and QAI efforts and a workshop with global experts who then participated in a process of iteratively identifying key items for consideration [4,5,8,17,22,23]. We will describe this background work briefly in subsequent sections and an overview is provided in Figure 1.

### 2.2 Research ethics review

This project was assessed by the Hamilton Integrated Research Ethics Board and determined to be quality improvement and exempt from a full review in accordance with institutional research ethics board policy.

### 2.3 Workshop and systematic review

We held a two-day workshop with global experts in guidelines and quality assurance scheme development in June 2018, sponsored by the European Commission [4]. To inform the workshop, we produced an evidence brief to provide a common background for participants on guidelines and quality assurance [24]. We described the workshop findings in a separate article [4]. Prior to that, we conducted a systematic review on guideline-based quality indicators that included grey literature such as manuals and other documents linking guidelines to QAI [5]. Finally, we clarified terminology and created a framework outlining the connections between guideline recommendation outcomes and quality indicators, performance measures, and performance indicators [8].

We used considerations identified in the systematic review to formulate an initial long-list of possible checklist items and the lead investigators (HJS, EP, ML, TP) iteratively refined and improved the list removing potential redundancies and ensuring comprehensiveness. We then presented the draft long-list of possible checklist extension items to attendees at a workshop [4]. Workshop participants included geographically diverse methodologists, guideline developers, QAI specialists, and consumer representatives, listed in Appendix 1 [4]. At the workshop, we held a structured discussion on the checklist extension and incorporated feedback from participants on the preliminary items.

### 2.4 Survey

We then conducted a feedback survey with workshop participants as respondents using the preliminary items identified in the systematic review, and organized into the existing GDC domains. The complete survey is available in Appendix 2. The survey was distributed to all workshop participants using Survey Monkey (surveymonkey.com). The survey presented each potential checklist extension item for QAI integrated with guidelines and asked participants to rate that item on the seven-point Likert scale (strongly disagree to strongly agree) with regards to its inclusion in the checklist extension. We summed the scores from −3 (strongly disagree) and +3 (strongly agree) for each participants' response corresponding to their Likert scale rating for each checklist item dividing by the maximum possible score (+3 × number of respondents) to generate a ‘percent agreeability’ from survey respondents for each item. We also collected open-ended feedback on each checklist item and to identify potential gaps or omissions from the extension.

### 2.5 Working group and refinement of checklist items

We then reviewed the results with a working group, comprised of nearly all workshop attendees and additional key experts (IK, OG), who initially reviewed the survey results. We collected item-level feedback from all working group members. The checklist was iteratively adapted by the working group, expanding, and consolidating through three phases of edits to arrive at the final checklist. We then iteratively sought feedback to refine and clarify specifically the characteristics of a good quality indicator as one of the steps in the checklist. At all meetings, we took detailed notes and reviewed changes with members electronically.

## 3 Results

### 3.1 Systematic review and preliminary checklist items

We initially proposed 43 items in the preliminary checklist extension (see Appendix 3). We linked these items back to the GIN-McMaster Guideline Development Checklist categories.

### 3.2 Workshop and survey

We received responses from all workshop attendees (*n* = 16). The full results of the survey are available in Appendix 3. Agreeability with each initial item ranged from 18.8% to 91.7%; nine survey participants provided qualitative feedback highlighting additional missing items, which informed additions to the checklist.

### 3.3 Working group and refinement of items

Wording edits and refinement were made to improve clarity were made immediately. For example, one contributor suggested for item number one “always add people to patient-important outcomes” so we changed this accordingly.

In other instances, useful details elaborating the original criteria were shared by the working group and discussed to determine inclusion. For example, for item 38, which originally read: “Consider pilot testing the quality indicators and performance measures with the target end-users (e.g., with members of target audience and stakeholders who participated in the development group)”, wording was added to further clarify: “The type of pilot testing may be different for different groups depending on the timeline and feasibility of an integrated guideline and quality assurance scheme, however, is a critical step for ensuring feasibility and implementation”.

Other items required significant further work and rewording including item 27, which originally read: “Use relevance, scientific soundness, feasibility, specification and intended use of performance measures as criteria to develop/define the quality indicator”. We elaborated this to be: “Consider the appropriateness of outcomes as quality indicators. ∗Appropriate quality indicators, as described in Table 1 should be: 1) High certainty in the quality indicator (evidence supporting it is at low risk of bias, precise, direct = relevant, consistent and without publication bias), in other words “scientifically sound”; 2) Responsive or sensitive to change (which may also be considered under risk of bias); 3) Feasible to measure, implement and monitor.”

**Table 1:** Characteristics of credible quality indicators

Characteristic	Operationalization
Certainty in the evidence	High certainty in the quality indicator (evidence supporting it is at low risk of bias, precise, direct = relevant, consistent and without publication bias), also described as “scientifically sound”
Measuring change	Responsive or sensitive to change (may also be considered under risk of bias)
Feasibility	Feasible to measure, implement and monitor

A final example of a change to the checklist was the addition of item number 36 based on working group feedback: “Propose mechanisms to document quality indicators or performance measures in a standardized (even anonymous) fashion to allow synthesis of data, collaboration and shared learnings across different health care systems and jurisdictions. This might provide a feedback mechanism for larger scale improvement and updating of guidelines, etc.”

In the third and final round of edits, we had achieved consensus with no suggestions for new items to add or remove were suggested and only minor proposed wording edits suggested. This checklist extension is intended to encompass all steps required to consider QAI schemes from a guideline-oriented perspective. The final checklist was composed of 40 items across the existing 18 domains of the GIN-McMaster Guideline Development Checklist and one additional domain specific to QAI [19]. It is available here: https://heigrade.mcmaster.ca/guideline-development/checklist-extension-for-QAI [19].

### 3.4 Characteristics of a proper quality indicator

While there is no international consensus on characteristics of good quality indicators, frameworks such as the RUMBA rule provide suggestions [25]. The RUMBA rule involves: “Relevant to the problem, Understandable, Measurable (with high dependability and validity), Behaviourable (changeable through behavior), Achievable and feasible” [25]. However, the RUMBA rule is not widely used in guideline-based quality indicators development and quality indicators often lacks a clear connection to the underpinning evidence and an assessment of the certainty of that evidence [6]. Our review of the RUMBA rule showed that the criterion of scientifi soundness requires further structure. We suggest that scientific soundness is equivalent to certainty in the evidence supporting the quality indicator. This certainy of the evidence can be operationalized through the GRADE certainty of evidence assessment. We therefore refined this characterisitic as certainty in the evidence supporting the quality indicator. We also identified responsiveness to change as another important characteristic that expresses that the quality indicator has the property of detecting change. This can feature can relate to risk of bias in the measurement of the quality indicator but it appears to be a separate domain. A result of our work is that to assess the certainty in the evidence of of a quality indicator, a detailed operationalization, through the GRADE domains, of the characteristics is required.

We discussed the likelihood that some indicators will be better in one characteristic or another. For example, in assessing quality indicators on breast cancer screening program, from the ECIBC, we there are two example quality indicators: ‘breast cancer incidence-based mortality’ and ‘screening test coverage’. Breast cancer incidence mortality as a quality indicator is a patient-important outcome with high certainty (characteristic 1); it is grounded in randomized control trial evidence and its measurement is scientifically sound (characteristic 1); it is very feasible to implement and monitor since it is already measured in cancer registry databases (characteristic 3); and if a screening program improves incidence mortality it is actioning improvement from a people-important perspective (characteristic 3). However, due to the long-time horizon anticipated in order to see changes in this quality indicator it may not be as sensitive to change (characteristic 2). Screening test coverage was also identified as a quality indicator. It is more sensitive to change (characteristic 2), however, because it is a surrogate measure for more important quality indicators such as incidence-based mortality, it may fail characteristic 1, if, for example, the screening program covers a large part of the population but with a low-quality mammography that misses cancers and leads to decrease in early diagnosis [26,27].

## 4 Discussion

In this article, we describe the process for the development of a checklist of items to be considered for integrated QAI and guideline development. The derived checklist is based on the GIN-McMaster Guideline Development Checklist. This work is premised on a systematic review, stakeholder engagement workshop and survey, and subsequent consensus process through iterative refinement of considerations for QAI. We previously found that methodological guidance to support QAI integration into guidelines is to date limited and that actual integrated approaches to guidelines and QAI are few [5]. In part, this can be attributed to the lack of appropriate guidance for accomplishing this challenging task. The result is a list of items that we hope will have practical use to guideline developers and individuals interested in improving evidence-based QAI schemes.

### 4.1 Strengths

A major strength of the present work is that this extension includes an applied and pragmatic list of considerations for the integration of QAI into guideline processes. The validity of this work comes from the use of a systematic review and incorporation expertise from both guideline and QAI experts in its development. Our extension format is also a strength: checklists are extensively used in guideline development as well as other fields and constitute a validated mechanism for ensuring reflection on and, if applicable, completion of critical steps. An additional strength is our efforts here to describe elements of a good quality indicator. Literature to date has been inconsistent and unclear on these characteristics, and through an iterative group process we have attempted to provide clarity to this critical definition.

### 4.2 Limitations

Firstly, as for any use of the GDC, we recognize that the list is long, and, importantly, that all steps may not be relevant to all integrated guideline and QAI efforts, but the intention is for the GDG to be comprehensive. Our work also has not been prospectively validated in an actual guideline, but this will be done in the ECICC. In addition, we were guideline-focused as opposed to QAI focused as the starting point. However, the expertise of the QAI experts and our systematic approach to identifying QAI should overcome some of these shortcomings. Furthermore, there are few concerted efforts for the integration of guidelines and QAI and thus, this work is novel. While giving the items in the checklist consideration is a straightforward task, the time requirements to conduct the identified steps are uncertain. For example, in countries such as the U.S where performance measurement is elaborate and often linked to remuneration schemes, it may not be feasible for guideline groups to address necessary steps to meet data gathering and validation needs within the short timeline that is often available to guideline groups. However, we assert that these challenges should not subvert rigorous methods and linkage to evidence in guideline-based QAI.

### 4.3 Implications for policy and practice

This checklist extension should be considered by guideline developers, including by guideline sponsor organizations which are seeking to improve the implementation, monitoring and evaluation of their guideline recommendations. Notably, guideline developers should define the scope of QAI linked to guidelines, gaps in existing QA and the perspective they will take at the outset. They should follow our clear terminology differentiating quality indicators, performance measures, and performance indicators, and utilize people-important outcomes linked to guideline recommendations to inform quality indicator/performance measure/performance indicator development [28]. Policy-makers should take note of steps identified in this checklist extension as key considerations to improve the planning and execution of QAI schemes linked to guidance. Encouragingly for this work and its implementation, recent research on reporting standards for performance measures linked to guidelines suggests these efforts can succeed in parallel or following guidelines with careful planning and attention to implementation [3]. Future iterations of the GIN-McMaster Guideline Developers Checklist will review and incorporate additional extensions as this tool becomes more comprehensive to improve the process and rigour of guideline development. For example, the checklist will be extended to address living reviews and stakeholder involvement in guideline development [18,29].

### 4.4 Implications for research

This checklist extension should be evaluated prospectively in integrated guideline and QAI projects. The forthcoming ECICC and the ongoing ECIBC will be two such projects [4,12]. We anticipate that this work will also support research regarding the implementation of guideline recommendations linked to QAI and its feasibility. Future research should validate the checklist in actual guideline projects to see if the items are logical and helpful to the guideline process. Future research should also assess the degree to which standardized methods, supported by a checklist, can improve the creation of QAI linked to guidelines that can ultimately improve health.

## 5 Conclusions

We have developed an extension of the GIN-McMaster GDC for QAI schemes. The principles we have incorporated should form the basis for integrated guidelines and QAI projects. Future work should evaluate which of the items require additional support for guideline developers and assess the impact of linked health guidelines and QAI schemes to help improve health systems and health outcomes.

## CRediT authorship contribution statement

**Thomas Piggott:** Conceptualization; Data curation; Formal analysis; Investigation; Methodology; Project administration; Resources; Software; Validation; Visualization; Roles/Writing–original draft; Writing–review & editing. Miranda Langendam: Conceptualization; Data curation; Formal analysis; Investigation; Methodology; Project administration; Resources; Validation; Visualization; Roles/Writing–original draft; Writing–review & editing. **Elena Parmelli:** Conceptualization; Data curation; Formal analysis; Investigation; Methodology; Project administration; Resources; Validation; Visualization; Roles/Writing–original draft; Writing–review & editing. **Jan Adolfsson:** Investigation; Methodology; Validation; Roles/Writing–original draft; Writing–review & editing. **Elie Akl:** Investigation; Methodology; Validation; Roles/Writing–original draft; Writing–review & editing. **David Armstrong:** Investigation; Methodology; Validation; Roles/Writing–original draft; Writing–review & editing. **Jeffrey Braithwaite:** Investigation; Methodology; Validation; Roles/Writing–original draft; Writing–review & editing. **Romina Brignardello-Petersen:** Investigation; Methodology; Validation; Roles/Writing–original draft; Writing–review & editing. **Jan Brozek:** Investigation; Methodology; Validation; Roles/Writing–original draft; Writing–review & editing. **Markus Follmann:** Investigation; Methodology; Validation; Roles/Writing–original draft; Writing–review & editing. **Ina Kopp:** Investigation; Methodology; Validation; Roles/Writing–original draft; Writing–review & editing. **Joerg J Meerpohl:** Investigation; Methodology; Validation; Roles/Writing–original draft; Writing–review & editing. **Luciana Neamtiu:** Investigation; Methodology; Validation; Roles/Writing–original draft; Writing–review & editing. **Monika Nothacker:** Investigation; Methodology; Validation; Roles/Writing–original draft; Writing–review & editing. **Amir Qaseem:** Investigation; Methodology; Validation; Roles/Writing–original draft; Writing–review & editing. **Paolo Giorgi Rossi:** Investigation; Methodology; Validation; Roles/Writing–original draft; Writing–review & editing. **Zuleika Saz-Parkinson:** Investigation; Methodology; Validation; Roles/Writing–original draft; Writing–review & editing. **Philip van der Wees:** Investigation; Methodology; Validation; Roles/Writing–original draft; Writing–review & editing. **Holger J. Schünemann:** Conceptualization; Formal analysis; Investigation; Methodology; Project administration; Resources; Supervision; Validation; Visualization; Roles/Writing–original draft; Writing–review & editing.
