# Supplementary material for: The GIN-McMaster guideline tool extension for the integration of quality improvement and quality assurance in guidelines: a description of the methods for its development
Source: J Clin Epidemiol. 2023 Feb;154:197–203. doi: 10.1016/j.jclinepi.2022.04.002 (PMC10109085; doi:10.1016/j.jclinepi.2022.04.002)
Supplement: Appendix 1 [file mmc1.docx]

**Appendix 1**. **Workshop Participants and Affiliated Organisations (Alphabetical Order)**

- Jan Adolfsson, Swedish Agency for Health Technology Assessment and Assessment of Social Services, Sweden
- Elie Akl, American University of Beirut, Lebanon
- David Armstrong, McMaster University, Canada
- Jane Beaumont, independent consultant for accreditation and certification
- Jeffrey Braithwaite, International Society for Quality in Healthcare (ISQua), Australia
- Romina Brignardello-Petersen, McMaster University, Canada
- Jan Brozek, McMaster University, Canada [online participation]
- Markus Follmann, German Cancer Society, Germany
- Paolo Giorgi Rossi, Inter-institutional Epidemiology Service, Reggio Emilia, Italy
- Jola Gore Booth, EuropaColon [patient representative], United Kingdom
- Zbigniew Les, EvidencePrime/GRADEpro, Poland
- Miranda Langendam, Department of Clinical Epidemiology, Biostatistics and Bioinformatives, University of Amsterdam
- Joerg J Meerpohl, Medical Center - University of Freiburg, Germany
- Susan Norris, World Health Organisation, Switzerland
- Luciana Neamtiu, European Commission, Joint Research Centre (JRC), Ispra, Italy
- Monika Nothacker, Institute of Medical Knowledge Management, Association of the Scientific Medical Societies (AWMF), Germany
- Elena Parmelli, European Commission, Joint Research Centre (JRC), Ispra, Italy
- Thomas Piggott, Department of Health Research Methods, Evidence, and Impact, McMaster University
- Amir Qaseem, American College of Physicians, United States of America
- Zuleika Saz-Parkinson, European Commission, Joint Research Centre (JRC), Ispra, Italy
- Holger Schünemann, Department of Health Research Methods, Evidence, and Impact, McMaster University
- Mariano Tomatis, Freelance IT specialist with a Degree in Computer Science working for hospital quality assurance systems
