# Supplementary material for: The GIN-McMaster guideline tool extension for the integration of quality improvement and quality assurance in guidelines: a description of the methods for its development
Source: J Clin Epidemiol. 2023 Feb;154:197–203. doi: 10.1016/j.jclinepi.2022.04.002 (PMC10109085; doi:10.1016/j.jclinepi.2022.04.002)
Supplement: Appendix 2 [file mmc2.pdf]

European Commission

ECICC Framework Quality Assurance Steps

CONSENT STATEMENT

Thank you for all of your work over the past three days here in Ispra!

You are being invited to participate in a survey to prioritize steps in the development of guideline-based quality assurance scheme for the forthcoming European Commission's Initiative on Colorectal Cancer (ECICC). This survey is part of an official methodological framework commissioned by the European Commission. Your input will inform the **development of the methodological framework** to prepare for the ECICC.

By completing this survey, you consent to your response being collected to be used for the methodological framework.
Disclaimer: While the ECICC will be informed by this survey, the **related mandate to eventually conduct the work on the guidelines and quality assurance scheme** will not be affected by the results of this survey.

Instructions:

We would like to obtain your views on the considerations and steps for guideline-based quality assurance scheme development. In completing the following questions, please consider the existing steps in guideline-development from the GIN-McMaster Guideline Development Checklist, which is available here:

http://heigrade.mcmaster.ca/guideline-development/gin-mcmaster-guideline-development-checklist/using-checklist

The steps that are recommended in this survey will inform additions to the GIN-McMaster Guideline Development Checklist for guideline-based quality assurance scheme development.

We realize that you may not be able to answer all questions or that you have more expertise in some than in other areas. Please do answer the questions to the best of your ability following your evaluation of the circulated feasibility plan. Should you have any questions or concerns please contact Dr. Holger Schünemann at holger.schunemann@mcmaster.ca or Dr. Miranda Langendam at m.w.langendam@amc.uva.nl.

It is estimated that this survey will take between 20-30 minutes to complete and will begin with questions about your background. With your consent, we may contact you to follow-up on any responses that we would like further clarification on.

Questions:

For each of the following NEW items please state your agreement (from strongly disagree to strongly agree) with their inclusion as part of a quality assurance extension to the Checklist.

*** 1. Organization, Budget, Planning and Training (although general here, it will be specifically based on the scope for the ECICC in the final framework)**

	Strongly Disagree	Disagree	Somewhat Disagree	Neither agree or disagree	Somewhat Agree	Agree	Strongly Agree

	Strongly Disagree	Disagree	Somewhat Disagree	Neither agree or disagree	Somewhat Agree	Agree	Strongly Agree
12. Identify all relevant input parameters from the different parts of the pathway to the overall patient important outcomes and quality indicators.							
13. Explore if outsourcing of specific tasks, e.g. systematic review conduct or quality assurance work, is required to conduct the work.							

*** 2. Priority Setting**

	Strongly Disagree	Disagree	Somewhat Disagree	Neither agree or disagree	Somewhat Agree	Agree	Strongly Agree
10. Identify gaps in accreditation and certification schemes on the topic.							
11. Search for quality indicators and performance measures on the topic.							
12. Identify the perspective that is taken (population, individual, health system)							
13. Consider where quality indicators should be assessed in relation to the evidence to decision-making process: parallel groups, integrated with the recommendations or sequential							

*** 3. Guideline Group Membership**

	Strongly Disagree	Disagree	Somewhat Disagree	Neither agree or disagree	Somewhat Agree	Agree	Strongly Agree
8. Determine if subgroups on specific topics are required and how they will interact with the larger group.							

*** 4. Establishing Guideline Group Processes**

	Strongly Disagree	Disagree	Somewhat Disagree	Neither agree or disagree	Somewhat Agree	Agree	Strongly Agree
6. Provide opportunities for discussion and feedback about the group process throughout the guideline development project. **But do not conduct methodological course corrections for approaches described in the operating procedures or guideline and quality assurance manual.**							
11. Develop a publication plan and authorship rules for any publications resulting from the work.							

*** 5. Identifying Target Audience and Topic Selection**

	Strongly Disagree	Disagree	Somewhat Disagree	Neither agree or disagree	Somewhat Agree	Agree	Strongly Agree

	Strongly Disagree	Disagree	Somewhat Disagree	Neither agree or disagree	Somewhat Agree	Agree	Strongly Agree
7. Decide if the evaluation of a quality indicator is an intervention question (e.g. Does the use of informed choice interventions compared to not using informed choice improve net consequences as opposed to using informed choice as a quality indicator of a conditional recommendation).							

*** 6. Consumer and Stakeholder Involvement**

	Strongly Disagree	Disagree	Somewhat Disagree	Neither agree or disagree	Somewhat Agree	Agree	Strongly Agree
10. Allow relevant stakeholders to be part of the consultation process.							

*** 7. Conflict of Interest (COI) Considerations**

	Strongly Disagree	Disagree	Somewhat Disagree	Neither agree or disagree	Somewhat Agree	Agree	Strongly Agree
8. Apply the same DOI and COI management rules to GL and QA (healthcare institutions representatives may require specific considerations).							
9. Consider credibility of the institution in declaring what is known to individuals and what is not known at the time of declaration.							

*** 8. (PICO) Question Generation**

	Strongly Disagree	Disagree	Somewhat Disagree	Neither agree or disagree	Somewhat Agree	Agree	Strongly Agree
1b. A logical model/analytical pathway/disease model/analytical PICO framework should be produced beginning with prevention to diagnosis to treatment to the outcomes.							
14. Determine if outcomes are feasible and measurable and important for people either directly or indirectly for quality assurance purposes.							
15. If the recommendation relates to the evaluation of a QI, then the group should consider using an intervention framework EtD to assess the QI (PICO/Topic Selection).							
16. Identify all relevant input parameters from the different parts of the pathway to the overall patient important outcomes and quality indicators							

*** 9. Considering Importance of Outcomes and Interventions, Values, Preferences and Utilities**

	Strongly Disagree	Disagree	Somewhat Disagree	Neither agree or disagree	Somewhat Agree	Agree	Strongly Agree

	Strongly Disagree	Disagree	Somewhat Disagree	Neither agree or disagree	Somewhat Agree	Agree	Strongly Agree
10. Rate or select a small but sufficient number of candidate QIs. Consider there relation to the people important outcomes and are valid long-term surrogates.							
11. Consider QIs that cannot be manipulated by those parties that are affected by them.							
12. When considering candidate outcomes as QI, describe their relevance, the supporting evidence (scientific soundness), and feasibility.							
13. Consider the precision of a QI.							

*** 10. Deciding what Evidence to Include and Searching for Evidence**

	Strongly Disagree	Disagree	Somewhat Disagree	Neither agree or disagree	Somewhat Agree	Agree	Strongly Agree
11. Evaluate if evidence supports that the use of a PM improves people outcomes.							
12. Check if there is evidence that certification and accreditation improves outcomes.							

*** 11. Summarizing Evidence and Considering Additional Information**

	Strongly Disagree	Disagree	Somewhat Disagree	Neither agree or disagree	Somewhat Agree	Agree	Strongly Agree
8. Consider items relevant for the development of decision aids.							
9. Reconsider what quality assurance or performance indicators can be developed.							

*** 12. Judging Quality, Strength or Certainty of a Body of Evidence (None Identified)**

*** 13. Developing Recommendations and Determining their Strength (including considerations about quality assurance)**

	Strongly Disagree	Disagree	Somewhat Disagree	Neither agree or disagree	Somewhat Agree	Agree	Strongly Agree
11. Consider which outcomes are measurable, feasible and relevant as quality indicators or performance measures							
12. Consider which outcomes are measurable, feasible, scientifically sound and relevant as quality indicators or performance measures etc. TO BE COMPLETED BAED ON CRITERIA.							

*** 14. Wording of Recommendations and of Considerations of Implementation, Research, Monitoring and Evaluation (including considerations about quality assurance)**

	Strongly Disagree	Disagree	Somewhat Disagree	Neither agree or disagree	Somewhat Agree	Agree	Strongly Agree
9. Select quality indicators and performance measures based on prioritized outcomes.	◯	◯	◯	◯	◯	◯	◯
10. Determine how the indicators will impact on accreditation and certification of organizations.	◯	◯	◯	◯	◯	◯	◯
11. Consider unintended consequences of QI on target population.	◯	◯	◯	◯	◯	◯	◯

*** 15. Preparation for quality assurance and selection of quality indicators**

	Strongly Disagree	Disagree	Somewhat Disagree	Neither agree or disagree	Somewhat Agree	Agree	Strongly Agree
1. Select quality indicators and performance measures based on prioritized outcomes.	◯	◯	◯	◯	◯	◯	◯
2. Determine how the indicators will impact on accreditation and certification of organizations.	◯	◯	◯	◯	◯	◯	◯
3. Use relevance, scientific soundness, feasibility, specification, intended use of performance measures as criteria to develop/define the QI.	◯	◯	◯	◯	◯	◯	◯
4. Consider which performance measures may be appropriate to be use with quality indicators.	◯	◯	◯	◯	◯	◯	◯
5. Ensure collaboration with those translating quality indicators and performance measures into performance indicators.	◯	◯	◯	◯	◯	◯	◯
6. Describe the intended use of the quality indicators and performance measures.	◯	◯	◯	◯	◯	◯	◯

*** 16. Reporting and Peer Review**

	Strongly Disagree	Disagree	Somewhat Disagree	Neither agree or disagree	Somewhat Agree	Agree	Strongly Agree
9. Develop or adopt a standardized format for describing the QA approach, with specific structure, headings, and content.	◯	◯	◯	◯	◯	◯	◯
10. Report in the monitoring and evaluation section the relevance of the QI, its face validity, scientific Soundness certainty of the evidence (including precision), feasibility, specify the QI, and its intended use. (and performance measure?)	◯	◯	◯	◯	◯	◯	◯

*** 17. Dissemination and Implementation (including considerations about quality assurance) - (None Identified)**

	Strongly Disagree	Disagree	Somewhat Disagree	Neither agree or disagree	Somewhat Agree	Agree	Strongly Agree
5. Consider which quality indicators may be used for certification and accreditation (e.g. those easy to measure and collect, already available, ready to benchmark)	◯	◯	◯	◯	◯	◯	◯
6. Determine what accountability mechanisms will be developed for the quality indicators.	◯	◯	◯	◯	◯	◯	◯

*** 18. Evaluation and Use (including considerations about quality assurance)**

	Strongly Disagree	Disagree	Somewhat Disagree	Neither agree or disagree	Somewhat Agree	Agree	Strongly Agree
7. Consider pilot testing the quality indicators and performance measures with the target end users (e.g. with members of target audience and stakeholders who participated in the development group).	○	○	○	○	○	○	○
8. Consider providing guidance on when to 'retire' or cease measuring performance measure.	○	○	○	○	○	○	○

*** 19. Updating**

	Strongly Disagree	Disagree	Somewhat Disagree	Neither agree or disagree	Somewhat Agree	Agree	Strongly Agree
7. Reevaluate the quality indicators, performance measures and performance indicators.	○	○	○	○	○	○	○

20. What additional feedback do you have regarding the steps in quality assurance scheme development? Please reference the checklist section that is relevant if possible.

*** 21. What is your name?**

First Name

Last Name

End of Questionnaire

Thank you very much for your participation in this survey and for your feedback on the checklist considerations.

Done
