# Supplementary material for: The GIN-McMaster guideline tool extension for the integration of quality improvement and quality assurance in guidelines: a description of the methods for its development
Source: J Clin Epidemiol. 2023 Feb;154:197–203. doi: 10.1016/j.jclinepi.2022.04.002 (PMC10109085; doi:10.1016/j.jclinepi.2022.04.002)
Supplement: Appendix 3 [file mmc3.docx]

### Appendix 3. Results of workshop survey for QAI extension of the GIN-McMaster guideline checklist

The percent agreement for each item (ranked by descending agreement) ranged from 18.75% to 91.67% (see table below).

The three items that had the highest agreement for inclusion were (*checklist category*): “Search for quality indicators and performance measures on the topic.” (*Priority Setting*); “Identify the perspective that is taken (population, individual, health system)” (*Priority Setting*); “Determine if subgroups on specific topics are required and how they will interact with the larger group.” (*Guideline Group Membership*).

The three items that had the lowest agreement were (*checklist category*): “Determine what accountability mechanisms will be developed for the quality indicators.” (*Dissemination and Implementation*); “Determine how the indicators will impact on accreditation and certification of organisations.” (*Preparation for quality assurance and selection of quality indicators*); “Consider credibility of the institution in declaring what is known to individuals and what is not known at the time of declaration.” (*Conflict of Interest (COI) Considerations*).

Results survey for agreement on QAI extension of GIN-McMaster checklist.

| Checklist Category | Item for Addition | % Agreement |
| --- | --- | --- |
| Priority Setting | 11. Search for quality indicators and performance measures on the topic. | 91.7% |
| Priority Setting | 12. Identify the perspective that is taken (population, individual, health system) | 81.3% |
| Guideline Group Membership | 8. Determine if subgroups on specific topics are required and how they will interact with the larger group. | 81.3% |
| Conflict of Interest (COI) Considerations | 8. Apply the same DOI and COI management rules to guideline and QA (healthcare institutions representatives may require specific considerations). | 81.3% |
| Establishing Guideline Group Processes | 11. Develop a publication plan and authorship rules for any publications resulting from the work. | 79.2% |
| Consumer and Stakeholder Involvement | 10. Allow relevant stakeholders to be part of the consultation process. | 79.2% |
| Organisation, Budget, Planning and Training (although general here, it will be specifically based on the scope for the ECICC in the final framework) | 12. Identify all relevant input parameters from the different parts of the pathway to the overall patient important outcomes and quality indicators. | 77.1% |
| Organisation, Budget, Planning and Training (although general here, it will be specifically based on the scope for the ECICC in the final framework) | 13. Explore if outsourcing of specific tasks, e.g. systematic review conduct or quality assurance work, is required to conduct the work. | 77.1% |
| Evaluation and Use (including considerations about quality assurance) | 8. Consider providing guidance on when to ‘retire’ or cease measuring performance measure. | 75.0% |
| Wording of Recommendations and of Considerations of Implementation, Research, Monitoring and Evaluation (including considerations about quality assurance) | 11. Consider unintended consequences of QI on target population. | 72.9% |
| Evaluation and Use (including considerations about quality assurance) | 7. Consider pilot testing the quality indicators and performance measures with the target end users (e.g. with members of target audience and stakeholders who participated in the development group). | 72.9% |
| Updating | 7. Re-evaluate the quality indicators, performance measures and performance indicators. | 72.9% |
| Considering Importance of Outcomes and Interventions, Values, Preferences and Utilities | 12. When considering candidate outcomes as QI, describe their relevance, the supporting evidence (scientific soundness), and feasibility. | 70.8% |
| Preparation for quality assurance and selection of quality indicators | 6. Describe the intended use of the quality indicators and performance measures. | 70.8% |
| (PICO) Question Generation | 14. Determine if outcomes are feasible and measurable and important for people either directly or indirectly for quality assurance purposes. | 68.8% |
| Preparation for quality assurance and selection of quality indicators | 3. Use relevance, scientific soundness, feasibility, specification, intended use of performance measures as criteria to develop/define the QI. | 68.8% |
| Reporting and Peer Review | 9. Develop or adopt a standardised format for describing the QA approach, with specific structure, headings, and content. | 68.8% |
| (PICO) Question Generation | 1b. A logical model/analytical pathway/disease model/analytical PICO framework should be produced beginning with prevention to diagnosis to treatment to the outcomes. | 66.7% |
| Preparation for quality assurance and selection of quality indicators | 1. Select quality indicators and performance measures based on prioritised outcomes. | 64.6% |
| Wording of Recommendations and of Considerations of Implementation, Research, Monitoring and Evaluation (including considerations about quality assurance) | 9. Select quality indicators and performance measures based on prioritised outcomes. | 62.5% |
| Preparation for quality assurance and selection of quality indicators | 5. Ensure collaboration with those translating quality indicators and performance measures into performance indicators. | 62.5% |
| Considering Importance of Outcomes and Interventions, Values, Preferences and Utilities | 10. Rate or select a small but sufficient number of candidate QIs. Consider there relation to the people important outcomes and are valid long-term surrogates. | 60.4% |
| Developing Recommendations and Determining their Strength (including considerations about quality assurance) | 11. Consider which outcomes are measurable, feasible and relevant as quality indicators or performance measures | 60.4% |
| Priority Setting | 13. Consider where quality indicators should be assessed in relation to the evidence to decision-making process: parallel groups, integrated with the recommendations or sequential | 56.3% |
| Deciding what Evidence to Include and Searching for Evidence | 11.   Evaluate if evidence supports that the use of a PM improves people outcomes. | 56.3% |
| (PICO) Question Generation | 16. Identify all relevant input parameters from the different parts of the pathway to the overall patient important outcomes and quality indicators | 54.2% |
| Dissemination and Implementation (including considerations about quality assurance) - (None Identified) | 5. Consider which quality indicators may be used for certification and accreditation (e.g. those easy to measure and collect, already available, ready to benchmark) | 54.2% |
| (PICO) Question Generation | 15. If the recommendation relates to the evaluation of a QI, then the group should consider using an intervention framework EtD to assess the QI (PICO/Topic Selection). | 52.1% |
| Considering Importance of Outcomes and Interventions, Values, Preferences and Utilities | 11. Consider QIs that cannot be manipulated by those parties that are affected by them. | 52.1% |
| Considering Importance of Outcomes and Interventions, Values, Preferences and Utilities | 13. Consider the precision of a QI. | 52.1% |
| Summarising Evidence and Considering Additional Information | 9. Reconsider what quality assurance or performance indicators can be developed. | 52.1% |
| Developing Recommendations and Determining their Strength (including considerations about quality assurance) | 12. Consider which outcomes are measurable, feasible, scientifically sound and relevant as quality indicators or performance measures etc. TO BE COMPLETED BAED ON CRITERIA. | 52.1% |
| Priority Setting | 10. Identify gaps in accreditation and certification schemes on the topic. | 50.0% |
| Identifying Target Audience and Topic Selection | 7. Decide if the evaluation of a quality indicator is an intervention question (e.g. Does the use of informed choice interventions compared to not using informed choice improve net consequences as opposed to using informed choice as a quality indicator of a conditional recommendation). | 47.9% |
| Deciding what Evidence to Include and Searching for Evidence | 12. Check if there is evidence that certification and accreditation improves outcomes. | 45.8% |
| Preparation for quality assurance and selection of quality indicators | 4. Consider which performance measures may be appropriate to be use with quality indicators. | 45.8% |
| Reporting and Peer Review | 10. Report in the monitoring and evaluation section the relevance of the QI, its face validity, scientific Soundness certainty of the evidence (including precision), feasibility, specify the QI, and its intended use. (and performance measure?) | 43.8% |
| Establishing Guideline Group Processes | 6.     Provide opportunities for discussion and feedback about the group process throughout the guideline development project. But do not conduct methodological course corrections for approaches described in the operating procedures or guideline and quality assurance manual. | 41.7% |
| Wording of Recommendations and of Considerations of Implementation, Research, Monitoring and Evaluation (including considerations about quality assurance) | 10. Determine how the indicators will impact on accreditation and certification of organisations. | 39.6% |
| Summarising Evidence and Considering Additional Information | 8. Consider items relevant for the development of decision aids. | 35.4% |
| Dissemination and Implementation (including considerations about quality assurance) - (None Identified) | 6. Determine what accountability mechanisms will be developed for the quality indicators. | 33.3% |
| Preparation for quality assurance and selection of quality indicators | 2. Determine how the indicators will impact on accreditation and certification of organisations. | 29.2% |
| Conflict of Interest (COI) Considerations | 9. Consider credibility of the institution in declaring what is known to individuals and what is not known at the time of declaration. | 18.8% |
| Judging Quality, Strength or Certainty of a Body of Evidence (None Identified) | n/a | n/a |
